# UCS Chaperone Folding of the Myosin Head: A Function That Evolved before Animals and Fungi Diverged from a Common Ancestor More than a Billion Years Ago

**DOI:** 10.3390/biom12081028

**Published:** 2022-07-26

**Authors:** Peter William Piper, Julia Elizabeth Scott, Stefan Heber Millson

**Affiliations:** 1 Department of Molecular Biology and Biotechnology, University of Sheffield, Sheffield S10 2TN, UK; 2 School of Life Sciences, University of Lincoln, Lincoln LN6 7DL, UK; 16662138@students.lincoln.ac.uk

**Keywords:** UCS proteins, She4, Hsp90, temperature stress, yeast, filamentous fungi

## Abstract

The folding of the myosin head often requires a UCS (Unc45, Cro1, She4) domain-containing chaperone. Worms, flies, and fungi have just a single UCS protein. Vertebrates have two; one (Unc45A) which functions primarily in non-muscle cells and another (Unc45B) that is essential for establishing and maintaining the contractile apparatus of cardiac and skeletal muscles. The domain structure of these proteins suggests that the UCS function evolved before animals and fungi diverged from a common ancestor more than a billion years ago. UCS proteins of metazoans and apicomplexan parasites possess a tetratricopeptide repeat (TPR), a domain for direct binding of the Hsp70/Hsp90 chaperones. This, however, is absent in the UCS proteins of fungi and largely nonessential for the UCS protein function in *Caenorhabditis elegans* and zebrafish. The latter part of this review focusses on the TPR-deficient UCS proteins of fungi. While these are reasonably well studied in yeasts, there is little precise information as to how they might engage in interactions with the Hsp70/Hsp90 chaperones or might assist in myosin operations during the hyphal growth of filamentous fungi.

## 1. The UCS Protein Function

Myosin molecules need to be subject to a very precise temporal and spatial chaperoning so that they acquire their affinity for actin in the proper context. This is directed, in part, by a chaperone dedicated to the folding of the myosin head, a protein with the characteristic UCS (UNC45, Cro1, She4) domain. This UCS chaperone function was initially identified through the study of *Caenorhabditis elegans Unc-45* (“UNCoordinated”) mutants, mutants that display defects in both motility [1,2] and cytokinesis during embryogenesis [3]. This led to the identification of a protein—UNC45—that associates with both Hsp90 and partially folded myosin [4]. The *C. elegans* UNC45 facilitates not just the folding of myosin, but also a regulation of myosin levels by targeting excess or damaged myosin to the proteasome for degradation [5]. It forms linear multimers, a filament assembly scaffold for a precise spatial organisation of the building blocks of myofilament formation and the organisation of sarcomeric repeats [6]. *Drosophila* studies have also highlighted the importance of the UCS protein function, both during late embryogenesis when the initial differentiation of cells into muscle tissue occurs, and at later stages of *Drosophila* development [7,8,9,10].

**Figure 1:**
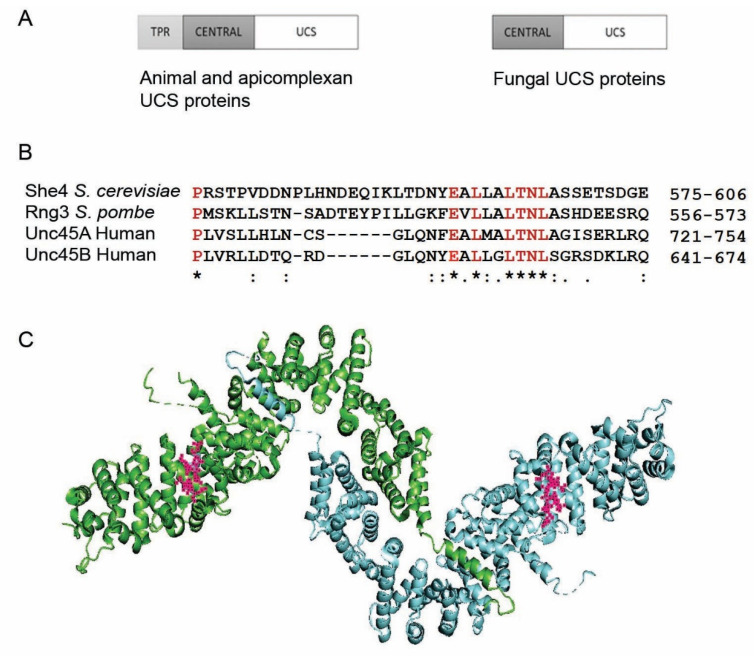
(**A**) Schematic diagram showing the domain structure of UCS proteins in animals and apicomplexan parasites (**left**) and fungi (**right**). (**B**) A small UCS sequence conserved from yeast to man. (**C**) The location (in red) of this **E**A**L**LA**LTNL** sequence in the two molecules within the unit cell of the X-ray crystal structure of She4, the UCS protein of the yeast *Saccharomyces cerevisiae* [17].

Except in fungi, UNC45 proteins have a 3-domain architecture [6,11] (Figure 1A). At their N-terminus is a tetratricopeptide repeat (TPR), a site for direct binding of the Hsp70 and Hsp90 molecular chaperones. This TPR is dispensable for UNC45 function in *C. elegans* [12] and zebrafish [13] and totally absent in the UCS proteins of fungi (Figure 1A). At the C-terminus, an elongated UCS domain mediates myosin folding, while a central domain aligns the TPR and UCS units to each other (Figure 1A). Direct biochemical proof that it is the UCS domain which mediates myosin folding came from demonstrations that the folding of muscle MHC-B myosin could be efficiently reconstituted in insect cells by the *C. elegans* UNC45, studies that revealed how the binding of the myosin substrate was compromised by the UCS domain mutations of temperature-sensitive *unc-45* mutants of *C. elegans* [14]. The central domain has been associated with a reversible inhibition of the myosin power stroke [15,16].

The UCS domain consists of repeats of an armadillo/beta-catenin-like motif, an approximately 40 amino acid-long sequence that was first identified in the *Drosophila* segment polarity gene armadillo and the mammalian armadillo homolog beta-catenin. The X-ray crystal structure of the *Drosophila* UNC45 reveals an L-shaped monomer in which a contiguous series of these armadillo repeats are stacked one upon another [7]. Self-association of these stacks causes UNC45 to exist as oligomers in vitro and in vivo [6,11], linear chains of UNC45 units that effectively form an assembly line for the licensing of the folding of myosin heads with a defined periodicity on myofilaments. How the conserved sequences of the flexible UCS interact with myosin is discussed in detail elsewhere [9,14,18].

## 2. Vertebrate Unc45A (UNC45-GC) and Unc45B (UNC45-SM)

While fungi, flies and worms have just a single UCS protein, vertebrates possess two, the latter denoted as Unc45A (or UNC45-GC) and Unc45B (or UNC45-SM) (reviewed in [9]). Unc45A is expressed in most somatic cells, where it acts upon non-muscle myosin II. Unc45B is expressed primarily in heart and skeletal muscle, where it facilitates the assembly and maintenance of the contractile apparatus [19,20]. Although largely not elaborated here, much attention is now being given to how an altered functioning of Unc45A and Unc45B might be associated with human genetic disorders [21,22,23,24,25].

Studies in zebrafish (*Danio rerio*) have revealed that Unc45A and Unc45B are not functionally redundant [26]. During *D. rerio* development, Unc45B is initially found in the myosin-containing A-band of the sarcomere. Later, in adult *D. rerio*, it is sequestered by the Z-lines in the mature sarcomere, though it is still able to shuttle back to the A-band of the muscle sarcomere in response to either eccentric exercise or damage induced by heat or chemical stress [7,27]. Both in zebrafish [13,26] and in the amphibian *Xenopus tropicalis* [28], the lack of a functional Unc45B results in paralysis, this being associated with loss of the thick and thin filament organisation of skeletal and cardiac muscle. Unc45B is also involved in eye development [29]. It appears essential that the levels of Unc45B should be precisely regulated, since a Unc45B overexpression in the skeletal muscles of zebrafish embryos causes defective myofibril organisation [13]; while in man a defective turnover of Unc45B is associated with hereditary inclusion-body myopathy, the affected individuals having severely disorganised myofibrils [25].

Unc45A is often elevated in tumour cells where it is thought to contribute to their proliferation and metastasis. In ovarian cancer, this elevated Unc45A is correlated with increases in cell motility and trafficked with its target myosin to the leading edges of the migrating cells [30]. Furthermore, Unc45A was recently found to break microtubules (MTs) independently of its effects on non-muscle myosin II and to destabilize MTs independently of its C-terminal UCS domain [31].

## 3. The UCS Function Evolved before Animals and Fungi Diverged from a Common Ancestor

The UCS chaperone function is generally considered vital for eukaryotic organisms though, as described below, this may not be the case for the yeast *S. cerevisiae*. Despite this, UCS proteins do not display the strong sequence conservation of many other molecular chaperones, such as those of the Hsp70/Hsp90 families. As shown in Figure 1B, a signature sequence central in the UCS domain has been remarkably conserved between the human Unc45A/B and the UCS proteins of fission yeast (*Schizosaccharomyces pombe*) and budding yeast (*S. cerevisiae*). The latter two yeast species diverged from each other more than 350 million years ago [32]. Furthermore, an expression of the human Unc45B—though not the human Unc45A—can provide partial rescue of the loss of UCS protein function in the yeast *S. cerevisiae* [33]. It is difficult to conduct meaningful phylogenetic analysis, such as has been done for myosins [34], on the basis of this short sequence alone in view of the considerable uncertainty as to whether any potential “hits” are functional UCS proteins.

## 4. Genetic Studies on the UCS Proteins of Ascomycete Fungi; UCS Function in the Absence of the TPR

Rng3, the sole UCS protein of fission yeast (*S. pombe*), has been shown to exist partly in association with polysomes [35]. This reveals that it binds co-translationally to the myosin heavy-chain polypeptides as the latter are synthesised de novo, prior to these myosin molecules acquiring their capacity for actin filament gliding. Compromised Rng3 action, as in certain conditional *RNG3* mutants of *S. pombe*, is associated with dramatically decreased levels of myosin and cortical actin patches, as well as a block to cytokinesis [36,37,38,39]. In *S. pombe* Rng3 is essential, as it is needed for the stabilisation of myosin II at the cytokinetic contractile ring [40].

**Figure 2:**
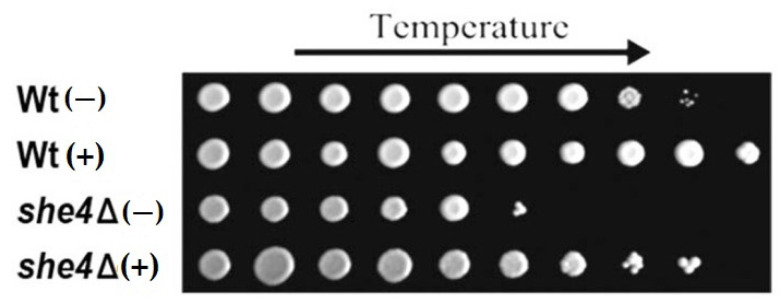
Wild type (Wt) and *she4Δ S. cerevisiae* cells pinned on 2% (*w*/*v*) peptone, 1% yeast extract, 2% glucose (YPD), 1.5% agar, and grown 2 days at 30 °C immediately following a prior 48 h growth on liquid YPD either without (−) or with (+) 1.2M sorbitol as osmotic stabiliser, this 48h growth having been conducted under 1.25 °C increases in temperature (left to right 30, 31.25, 32.5, 33.75, 35, 36.25, 37.5, 38.75. 40 and 41.25 °C).

While *S. pombe* has two myosin II species (Myo2 and Myp2), budding yeast (*S cerevisiae*) has just one (Myo1). Furthermore, cytokinesis in *S. pombe* requires both the catalytic and tail domains of this myosin II, while in *S. cerevisiae* just the tail of the sole myosin II (Myo1) can support cytokinesis [41]. This may explain why the UCS protein of *S. cerevisiae* (She4) is nonessential under many conditions of growth, unlike Rng3 of *S. pombe*. The *she4Δ* *S. cerevisiae* gene deletant is normally moderately temperature-sensitive, but its defective growth at high temperatures is substantially rescued by the osmotic stabilisation of the medium (Figure 2). Thus, while the UCS chaperone is widely considered to provide a critical function in eukaryotic organisms, this appears not to be the case for osmotically stabilised budding yeast.

In *S. cerevisiae* She4 acts on the two myosin-I forms (Myo3 and Myo5) and one of two myosin-V isoforms (Myo4) so as to enhance their folding and to reduce their turnover [39,42]. Its function is evidently more important as temperature is increased, since the phenotypes of the *S. cerevisiae she4Δ* mutant are most marked at higher temperatures. At 37–39 °C, *she4Δ* mutant cells exhibit severe defects in the organisation of the actin cytoskeleton (a functional Myo5-green fluorescent protein (GFP) fusion becoming dispersed through the cytosol and displaying an almost total loss of patch-like localisation to actin cortical patches), as well as defective endocytosis (apparent from a relatively weak FM4-64 staining of the vacuole) [42,43,44,45,46]. At slightly higher temperatures (45 °C), these *she4Δ* cells lyse [46]. It is still unclear why the loss of She4 should lead to a defect in cell wall integrity at high temperature (Figure 2). Cells of the *she4Δ* mutant are also defective in mating-type switching during haploid cell divisions, a reflection of the requirement for She4 in the formation of the functional cytoskeleton that can allow the asymmetric localisation of *ASH1* mRNA to daughter cells [47].

The filamentous ascomycete *Podospora anserina* is yet a third fungus in which the UCS protein function has been studied [48]. In this species, it is essential for sexual reproduction, the defective UCS function of the *cro1-1* mutant causing fruiting bodies to contain few asci and giant plurinucleate cells instead of dikaryotic cells after fertilisation. Karyogamy is not impaired, but the resultant polyploid nuclei generally undergo abortive meiosis, the *cro1-1* mutant being compromised in its inability to form septa between the daughter nuclei after each mitosis [48].

## 5. Myosins in Fungal Growth

In the budding yeast *S. cerevisiae,* a short period of polarised apical growth is followed by an extended isotrophic growth. The latter allows for the delivery of cell wall material over the entire bud surface, thereby leading to an almost spherical daughter cell. In contrast, filamentous fungi generally form hyphae that consist of chains of elongated cells that expand at the apex of the tip cell. During hyphal tip growth, cytoplasmic expansion forces are thought to push the cytoplasm against the flexible apical wall to power the expansion of the plastic apex. Hyphal extension involves the long-distance, polar delivery of Golgi-derived exocytic transport vesicles to this hyphal tip by MT-based kinesin motors (kinesins are not present in *S. cerevisiae*). At the hyphal apex, the fibres of the cell wall, such as chitin or glucan chains, are also synthesised, but as they are not yet cross-linked, the wall is still flexible at this point. Then, as the tip expands, the subapical chitin crystallises and becomes covalently bound to β-1,3-glucans, thereby solidifying the cell wall in the older parts of the growing hyphae.

At the hyphal apex, a forward-moving structure termed the Spitzenkörper determines the direction and rate of hyphal growth. Besides being the destination of exocytic transport vesicles, it also plays a role in endocytosis and membrane recycling (reviewed in [49]). Hyphal tip growth requires not just Spitzenkörper-directed polarised exocytosis at the expanding cell tip, but also the F-actin- and myosin-based transport of secretory vesicles along microfilaments. Actin-binding formin proteins anchor actin filaments to the growing tip and support actin assembly at the plus ends (barbed end) of these actin filaments.

Studies in *S. cerevisiae* [50,51,52] and *S. pombe* [53,54,55] have revealed that it is myosin-V motors that move exocytic vesicles towards the F-actin plus ends at plasma membrane regions of growth, whereas myosin-I motors support endocytosis [56]. A similar situation appears to apply in filamentous fungi. In *Aspergillus nidulans,* myosin-V interacts with vesicle transport proteins [57], while in the plant pathogen *Ustilago maydis*, a functional myosin-V-GFP fusion localises to the apical dome of hyphae [58]. In both *A. nidulans* [59,60,61] and *Candida albicans* [62], myosin-I is essential for hyphal growth and the endocytotic uptake of the endocytic marker dye FM4-64 into the vacuole [61,62]. Interestingly, a mutant form of the *A. nidulans* myosin-I that is almost devoid of ATPase activity can still support hyphal growth, indicating that myosin-I does not “walk” along actin filaments to mediate endocytosis [63]. One can surmise that UCS proteins are probably critical for these myosin-I and myosin-V operations in fungal hyphae, but in the absence of hard data this is still conjecture.

## 6. Hsp90 in UCS Protein Function

Pioneering in vitro studies on the folding of the myosin motor domain first revealed that mouse Unc45A and Unc45B can both dramatically enhance the Hsp90-dependent folding of a smooth muscle myosin motor domain-GFP fusion, Unc45A being more effective than Unc45B in this regard [64]. Striated muscle Unc45B was also shown to form a stable complex with Hsp90, a complex that selectively bound the partially folded conformation of the myosin motor domain synthesised in a reticulocyte lysate [65].

Unc45A and Unc45B differ in their associations with Hsp90α and Hsp90β, the two forms of cytosolic Hsp90 in vertebrate cells [66]. In many tissues, it is Hsp90β that is expressed constitutively at a high level, whereas Hsp90α is induced primarily in response to stress [66]. These two isoforms of Hsp90 have some distinct functional roles. In mice, Hsp90β [67] is essential for embryonic development [68,69], while a total loss of Hsp90α is fully compatible with viability but causes a block to spermatogenesis [70]. Zebrafish Hsp90α is highly expressed in striated muscle [67], its selective association with Unc45B being essential for the skeletal muscle organisation of embryos [68]. In contrast, it is Hsp90β and Unc45A that predominate in the other tissues of zebrafish [69]. These apparent preferences of Hsp90α for Unc45B and of Hsp90β for Unc45A are an indication of an evolutionary divergence of the respective Hsp90/UCS systems for the folding of non-muscle myosins versus cardiac and skeletal muscle myosins.

Except in fungi, UCS proteins have a TPR domain for direct interaction with the Hsp70/Hsp90 chaperones. Hsp90/Hsp70 binding by *C. elegans* UNC45 is abolished with the loss of this TPR [14]. Nevertheless, an expression of the UCS of this UNC45 alone can rescue *unc-45* null mutants of *C. elegans* arrested in embryonic muscle development, revealing the TPR to be dispensable for UNC45 function in vivo [12]. Tantalisingly, it is thought that the TPR/Hsp90 interaction may be actually inhibitory for the action of UNC45 since titration experiments show that, on a per molecule basis, the UCS alone has a greater activity in vivo in *C. elegans* muscle than the full-length UNC45 protein [12]. Also in zebrafish, loss of the TPR domain of Unc45B has no disruptive effect on myosin thick filament organisation [13]. This Unc45B of zebrafish undergoes an Hsp90-independent interaction with a protein-Apo2 that is required for the integrity of the myosepta and myofiber attachment [71].

Despite the absence of a TPR domain in the UCS proteins of fungi, there is evidence that the latter still associate with Hsp90 although the precise molecular details of these interactions remain unresolved. The *S. pombe* Rng3 binds Hsp90, loss of this interaction being suggested as the reason that a temperature-sensitive mutant of fission yeast Hsp90 (swo1-w1) is defective in actomyosin ring assembly at the restrictive temperature [38]. Certain temperature-sensitive Hsp90 mutants of *S. cerevisiae* also display a defective Myo5-GFP localisation (S.H.M., unpublished). The interactions of the *S. cerevisiae* She4 in the yeast two-hybrid system reveal that in vivo the Hsp90-She4 interaction strengthens dramatically as temperature is raised [46,72,73]. This may be correlated with She4 having a much more prominent role in *S. cerevisiae* at higher temperatures, as mentioned above. Elevated temperature acting to reinforce the Hsp90-She4 interaction might be a consequence of the UCS domain undergoing dramatic topology changes as temperature is increased, as previously observed for the UCS domain of Unc45B [74]. It may also reflect Hsp90/UCS interaction being required, not just for the assembly of a cytoskeleton, but also for the actions of Hsp90 and UFD-2 (ubiquitin fusion degradation 2) in repair of the myofibrillar disorganisation of stress [75].

## 7. Conclusions

Computational phylogenetics has revealed that fungi are more closely related to animals than plants, with animals and fungi diverging from a common ancestor more than a billion years ago [76]. The conservation of UCS domain structure—animals to fungi—(Figure 1A) suggests that the UCS function evolved prior to this divergence, possibly at the same time as a primordial myosin. The TPR may have been lost subsequently in fungi, as it is still present in the UCS proteins of the apicomplexan parasites *Toxoplasma gondii* and *Plasmodium falciparum* [77]. Apicomplexans are—based on small subunit ribosomal RNA sequencing—older than the three multicellular kingdoms of animals, plants, and fungi.

In this article we highlight the paucity of knowledge as to UCS protein function in fungi, apart from yeasts. The earliest fungi were unicellular marine, flagellated organisms [78]. Animals and fungi both possess uniflagellated reproductive stages (the sperm of animals and the zoospores of chytrid fungi). Flagellar movement is MT-based rather than myosin-dependent, but it is noteworthy that Unc45A was recently found to destabilise MTs in human and rat cells [31], indicating that UCS proteins may influence the functioning of MTs in other species as well. Some unicellular organisms can switch between a flagellar motility and an amoeboid motility [79]. While amoeboid motility is generally considered an animal cell property, it would appear not to have been totally lost in fungi, as it is apparent in a mutant *Neurospora crassa* which is defective in the synthesis of the (1,3)-β-d-glucan needed for cell wall assembly and which cannot form hyphae [80].

Multiple activities contribute to the expression, folding, assembly and interplay of actin and myosin, as well as in maintaining the functionality of actomyosin filaments during situations of stress. While UCS proteins are key in this regard, their interplay with many of the other chaperones and activities for protein turnover is still poorly understood. Screens have identified a number of other chaperones required for muscle integrity in *C. elegans*, including CeHop, CeAha1 and Cep23 [81]. Enabling Hsp70/Hsp90, their accessory components and the systems for protein turnover to establish and maintain the intricate myosin-actin interplay clearly presents a major challenge for the cellular chaperone machinery.

## Data Availability

N/A, review article.
